# Cholesterol, Triglyceride, and Glucose Levels Across Birth Cohorts in the US

**DOI:** 10.1001/jamanetworkopen.2024.49481

**Published:** 2024-12-06

**Authors:** Xiaoning Huang, Lucia C. Petito, Nilay S. Shah, Donald M. Lloyd-Jones, Sadiya S. Khan, Natalie A. Cameron

**Affiliations:** 1 Division of Cardiology, Department of Medicine, Northwestern University Feinberg School of Medicine, Chicago, Illinois; 2 Department of Preventive Medicine, Northwestern University Feinberg School of Medicine, Chicago, Illinois; 3 Division of General Internal Medicine, Department of Medicine, Northwestern University Feinberg School of Medicine, Chicago, Illinois

## Abstract

**Importance:**

Population mean levels of total cholesterol and fasting triglycerides have decreased substantially in the US in recent decades, but improvements in cardiometabolic health may have slowed among younger cohorts. Conversely, prevalence of diabetes (types 1 and 2) and obesity has increased, especially among adults younger than 65 years. It is unclear how trends in cholesterol, triglyceride, and glucose levels have changed across different birth cohorts and whether adverse trends in obesity are associated with these patterns.

**Objective:**

To quantify national trends in total cholesterol, fasting triglyceride, and fasting glucose levels among cohorts born between 1920 and 1999 and examine the potential association of these patterns with body mass index (BMI).

**Design, Setting, and Participants:**

This serial cross-sectional study used National Health and Nutrition Examination Survey (NHANES) data from the 1999-2000 to 2017-2020 cycles. Data were analyzed between November 1, 2023, and July 31, 2024. Participants included nonpregnant and noninstitutionalized US adults 18 years or older, born between 1920 and 1999, who had data collected from 1999 to 2020.

**Exposure:**

Eight 10-year birth cohorts (from 1920 to 1999).

**Main Outcomes and Measures:**

Total cholesterol, fasting triglyceride, and fasting glucose levels and BMI. Quantile regression models reported average marginal effects to quantify mean change in cardiometabolic outcome measures per decade of birth years. Parametric regression models estimated the association of birth cohort with outcomes, assessing BMI as the mediator.

**Results:**

Of 52 006 participants weighted to represent 264 664 915 US adults, weighted median age was 46 (IQR, 33-60) years and 50.6% were women. For the 50th percentile of measures, mean difference per 1-decade younger birth cohort was −7.1 (95% CI, −8.2 to −6.1) mg/dL for total cholesterol level, −13.1 (95% CI, −15.1 to −11.1) mg/dL for fasting triglyceride level, and 2.7 (95% CI, 2.3-3.1) mg/dL for fasting glucose level. BMI appeared to attenuate the associations between birth cohort and lipid levels and enhanced the associations between birth cohort and fasting glucose levels. However, up to 80% of the associations between birth cohorts and cardiometabolic outcomes were not mediated through BMI.

**Conclusions and Relevance:**

In this cross-sectional study of 52 006 participants representing 264 664 915 US adults, population-level improvements in total cholesterol and triglyceride levels decelerated and adverse trends in glucose levels accelerated in more recent birth cohorts, which was partially mediated by concurrent increases in BMI. Public health initiatives that target antecedent health behaviors are needed to improve cardiometabolic health across generations.

## Key Points

**Question:**

Have total cholesterol, fasting triglyceride, and fasting glucose levels changed across US birth cohorts, and is body mass index (BMI) associated with these patterns?

**Findings:**

In this cross-sectional analysis of 52 006 participants weighted to represent 264 664 915 US adults born between 1929 and 1999, total cholesterol and fasting triglyceride levels were lower, while fasting glucose levels and BMI were higher in more recent birth cohorts. Up to 80% of the associations between birth cohorts and cardiometabolic outcomes were not mediated through BMI.

**Meaning:**

These findings suggest that public health initiatives targeting antecedent health behaviors are needed to improve cardiometabolic health across generations.

## Teaser

This cross-sectional study describes US trends in total cholesterol, triglyceride, and glucose levels across birth cohorts and quantifies the degree to which these birth cohort effects may be mediated by population shifts in body mass index.

## Introduction

Cardiometabolic diseases, including coronary heart disease, heart failure, and diabetes (types 1 and 2), affect over 28 million people in the US. Although cardiometabolic-related death rates declined from the 1970s to the 2010s, favorable trends in death rates have since reversed.^1^ Understanding the prevalence and trends in antecedent risk factor levels are key to guiding public health and policy changes to reduce the population burden of cardiometabolic disease. Laboratory-based testing for levels of cholesterol, triglycerides, and glucose is routinely performed in clinical practice to evaluate and treat cardiometabolic risk.^2^ Mean total cholesterol and triglyceride levels have decreased substantially in the US over the past several decades, in the setting of higher levels of statin use and trans-fat regulation policies.^3,4^ However, emerging data suggest that improvements have been relatively slower among younger populations.^4,5^ Conversely, diabetes prevalence has increased by approximately 30% from 2001 to 2018, with more rapid increases observed among people younger than 65 years.^4,6,7,8^ Differing trends by age suggest the presence of population-level factors that may be uniquely influencing cardiovascular disease risk factors among generations as they move across time, also known as birth cohort effects.^4^

During this same time frame, obesity has been a growing epidemic in the US, and continues to be a major public health issue with rising rates in all age groups, especially younger adults.^9^ According to the National Health and Nutrition Examination Surveys (NHANES), the age-adjusted obesity prevalence among US adults increased from 31% in 1999 to 42% in 2020.^10^ Some of the most common risk factors for cardiometabolic diseases, including dyslipidemia and type 2 diabetes, can be directly attributed to obesity.^11^ However, the degree to which obesity, as measured by body mass index (BMI), has contributed to patterns in cholesterol, triglyceride and glucose values across time and generations is unclear. Therefore, this study aimed to (1) describe national trends in levels of total cholesterol, triglycerides, and glucose across birth cohorts and (2) quantify the degree to which these birth cohort effects may be mediated by population shifts in BMI.

## Methods

### Data Source and Study Population

We conducted a serial, cross-sectional analysis using NHANES data from nine 2-year survey cycles from 1999 to 2016, and a pre–COVID-19 pandemic cycle from 2017 to March 2020. NHANES data were collected and collated by the National Center for Health Statistics–Centers for Disease Control and Prevention, which used complex, multistage area probability sampling to select a nationally representative sample of noninstitutionalized US adults.^12^ Data regarding health-related factors and behaviors were collected at mobile examination centers (MECs) via in-person interviews, physical examinations, and nonfasting laboratory assessments, including total cholesterol level. A subset of participants was randomly assigned to a morning examination session to obtain fasting laboratory measurements, including triglyceride and glucose levels.^12,13,14^

**Figure 1.**
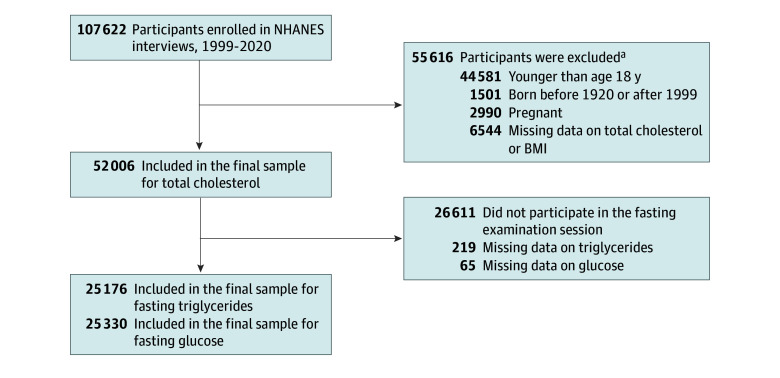
Sample Inclusion and Exclusion Flowchart BMI indicates body mass index; NHANES, National Health and Nutrition Examination Survey. ^a^Participants could be excluded for more than 1 reason.

We included nonpregnant MEC participants 18 years or older who were born between 1920 and 1999. For analyses of trends in glucose and triglyceride levels, eligible participants were those who attended the fasting examination. People with missing data on lipid or glucose levels were also ineligible (Figure 1). NHANES participants provided written informed consent. This analysis was exempt from Northwestern University’s institutional review board approval due to the use of deidentified, public-release data. The reporting of results adheres to the Strengthening the Reporting of Observational Studies in Epidemiology (STROBE) guidelines.

### Demographic and Birth Cohort Measurements

Data regarding age, sex, and race and ethnicity (Hispanic, non-Hispanic Asian, non-Hispanic Black, non-Hispanic White, and other) were self-reported at in-person NHANES interviews. Race and Hispanic origin were asked separately in the questionnaire but combined into a single variable in the published NHANES data. In the published data, American Indian or Alaska Native, Asian, multiracial, other, “don’t know,” and declined to answer were combined into the category of “other.” We further combined Mexican American and other Hispanic groups into a single Hispanic group. Year of birth was calculated by subtracting participant age from the survey year. Similar to previous publications, 8 birth cohorts were defined in 10-year intervals: 1920 to 1929, 1930 to 1939, 1940 to 1949, 1950 to 1959, 1960 to 1969, 1970 to 1979, 1980 to 1989, and 1990 to 1999.^15,16^

### Laboratory and Anthropomorphic Measurements

Outcomes of interest included measures of total cholesterol, fasting triglyceride, and fasting glucose levels. Venous blood samples were collected and frozen in MECs, then shipped to affiliated laboratories for analysis. All outcomes were measured via direct enzymatic assay and are reported in milligrams per deciliter. A subgroup of participants completed a morning blood draw after fasting between 8.5 and 24 hours for assessment of plasma triglyceride and glucose levels. Although modifications to laboratories, collection methods, and instruments occurred across survey periods, NHANES uses the Lipid Standardization Program of the Centers for Disease Control and Prevention to standardize serum lipid measurements to ensure accuracy and precision of measurements between laboratories across surveys.^13,17,18^ The non–high-density lipoprotein (HDL) cholesterol level has been recognized as a better predictive factor associated with cardiovascular disease risk.^19^ However, NHANES changed the HDL cholesterol assessment method since the 2003-2004 cycle, which would affect the calculation for non-HDL cholesterol. Thus, we used total cholesterol level as the main outcome for better consistency over survey cycles. Non-HDL cholesterol level was included as a secondary outcome, and results are presented in eTable 2 in Supplement 1.

Height and weight were measured by trained health technicians in the MEC using a standard stadiometer and digital weight scale, respectively.^20^ BMI was calculated as the weight in kilograms divided by the height in meters squared and rounded to the nearest tenth.

### Statistical Analysis

First, unadjusted 90th, 75th, 50th, 25th, and 10th percentiles of total cholesterol, triglyceride and glucose levels were calculated for the overall sample and for each 10-year birth cohort. Adjusted values were determined using multivariable, quantile regression models. The models used the continuous birth cohort as the main predictive factor associated with outcome and further adjusted for continuous age, sex, and self-reported race and ethnicity (using non-Hispanic White race as the reference category), as well as age^2^ and birth cohort^2^ to allow for curvilinear associations among age, birth cohort, and outcome variables. Similar specifications were used in previous literature.^15,21^ Race and ethnicity are social constructs that represent participants’ social determinants of health and were included in the model as a potential confounder because the different racial and ethnic distribution across birth cohorts could be associated with the outcomes of the study. An age-by-birth cohort interaction term was tested to assess whether the associations between birth cohort and outcomes differ by age (eFigure in Supplement 1). We used quantile regression and reported the average marginal effects (AMEs) to estimate the overall birth cohort effect, which was represented as the expected change in the outcomes associated with a marginal change (approximately equal to a 1-unit change in birth year, which is a decade) in birth year,^22^ holding the covariates constant. Positive AMEs (eg, birth cohort effects) indicate higher levels in the percentiles of the outcomes among relatively younger cohorts. We used quantile regression for 2 main reasons. First, the distributions of the cardiometabolic outcomes were highly skewed. Second, individuals with higher percentiles of lipid and glucose outcomes are much more likely to be treated pharmacologically. Using quantile regression, we were able to quantify cohort trends within each percentile. Cohort trends within lower percentiles of glucose and lipid outcomes were less likely to be influenced by changes in prescribing practices.

Given the nonlinear association observed between birth cohort and outcomes, we additionally used binary birth cohort variables to estimate marginal effects for each 10-year interval birth cohort (eg, 1930s vs 1920s, 1940s vs 1930s). We did not find significant interaction between age and cohort in the regression analysis and therefore dropped the interaction term from the mediation analysis.

In supplementary analysis, to account for the potential influence of statin use on cohort trends, we additionally adjusted for self-reported statin use in all regression and mediation models. We also tested the interaction between birth cohort and a binary time variable (before or after 2013) to determine whether the birth cohort effects were associated with the 2013 change in the guideline for therapy to lower lipid levels.

To estimate the association of BMI with birth cohort effects, we used causal mediation analysis with parametric regression models.^23^ This approach statistically strengthens the potential causal implications by reducing the bias created by unmeasured confounding of the exposure-outcome, mediator-outcome, or exposure-mediator associations.^24,25,26,27^ The method estimates 3 quantities: the natural direct effect, the natural indirect effect (ie, mediated effect), and the total effect. Briefly, the natural direct effect captures the effect of exposure (birth cohort) on the outcome (total cholesterol, triglyceride, or fasting glucose levels) in the absence of the mediator (BMI). The indirect pathway is the effect of the exposure on the outcome through the mediator. The total effect is the sum of the direct and indirect effects.

NHANES MEC weights were incorporated in the quantile regression analyses for total cholesterol level, and fasting subsample weights were used for triglyceride and glucose levels to improve the representativeness of the estimates. All analyses were conducted using Stata, version 17 (StataCorp LLC).^28^ The causal mediation analysis program does not support use of weights. Two-sided *P* < .05 indicated statistical significance. All analyses were performed between November 1, 2023, and July 31, 2024.

## Results

Of 52 006 nonpregnant adults in the primary analytic sample (weighted sample of 264 664 915 adults), the weighted median age was 46 (IQR, 33-60) years; 133 920 447 (50.6%) identified as female and 130 744 468 (49.4%) as male. For race and ethnicity, 37 847 083 adults (14.3%) identified as Hispanic, 28 319 146 (10.7%) as non-Hispanic Black, 178 913 482 (67.6%) as non-Hispanic White, and 19 585 204 (7.4%) as other (Table 1). Older birth cohorts had a larger proportion of female participants and were more likely to identify as non-Hispanic White. The weighted distributions of participants by birth cohort and age are shown in eTable 1 in Supplement 1. The quantile regression models showed that for adjacent cohorts with overlapping ages, there were decreases in total cholesterol and triglyceride levels in more recent cohorts (eFigure in Supplement 1). These trends were consistent across all birth cohorts from 1920s to 1990s.

**Table 1.** Weighted Sample Demographic Characteristics Overall and by Birth Cohorts: NHANES 1999-2020^a^

Characteristic	Birth cohorts, weighted No. (%)								
	1920-1929 (n = 2119)	1930-1939 (n = 5347)	1940-1949 (n = 7193)	1950-1959 (n = 8462)	1960-1969 (n = 8705)	1970-1979 (n = 8071)	1980-1989 (n = 8446)	1990-1999 (n = 3663)	All (N = 52 006)
Equivalent No. of US adults	5 925 664	16 258 597	33 123 071	47 102 291	52 511 476	45 158 816	41 155 609	23 429 390	264 664 915
Age, median (IQR), y	79 (76-80)	73 (69-79)	67 (60-73)	56 (50-62)	47 (41-53)	38 (31-43)	29 (24-34)	23 (21-26)	46 (33-60)
BMI, median (IQR)	27 (24-30)	28 (25-31)	28 (25-32)	28 (25-33)	28 (25-33)	28 (24-32)	27 (23-32)	26 (22-32)	28 (24-32)
Sex									
Female	3 537 621 (59.7)	9 137 332 (56.2)	17 753 966 (53.6)	24 022 169 (51.0)	26 623 318 (50.7)	22 172 979 (49.1)	19 590 070 (47.6)	11 175 819 (47.7)	133 920 447 (50.6)
Male	2 388 043 (40.3)	7 121 266 (43.8)	15 369 105 (46.4)	23 080 123 (49.0)	25 888 157 (49.3)	22 985 837 (50.9)	21 565 539 (52.4)	12 253 571 (52.3)	130 744 468 (49.4)
Race and ethnicity									
Hispanic	278 506 (4.7)	1 121 843 (6.9)	2 649 846 (8.0)	4 663 127 (9.9)	7 351 607 (14.0)	8 805 969 (19.5)	8 066 499 (19.6)	4 873 313 (20.8)	37 847 083 (14.3)
Non-Hispanic Black	373 317 (6.3)	1 268 171 (7.8)	2 682 969 (8.1)	4 945 741 (10.5)	5 828 774 (11.1)	5 057 787 (11.2)	4 979 829 (12.1)	3 116 109 (13.3)	28 319 146 (10.7)
Non-Hispanic White	5 131 625 (86.6)	13 267 015 (81.6)	25 769 749 (77.8)	34 478 877 (73.2)	35 550 269 (67.7)	27 366 242 (60.6)	24 158 343 (58.7)	13 120 459 (56.0)	178 913 482 (67.6)
Other^b^	142 216 (2.4)	601 568 (3.7)	2 020 507 (6.1)	3 014 547 (6.4)	3 780 826 (7.2)	3 883 658 (8.6)	3 950 938 (9.6)	2 319 510 (9.9)	19 585 204 (7.4)
Statin use	1 232 538 (20.8)	5 609 216 (34.5)	11 791 813 (35.6)	11 069 038 (23.5)	6 458 912 (12.3)	1 761 194 (3.9)	493 867 (1.2)	23 429 (0.1)	38 376 413 (14.5)

Abbreviations: BMI, body mass index (calculated as the weight in kilograms divided by the height in meters squared); NHANES, National Health and Nutrition Examination Survey.

^a^ Owing to weighting, numbers do not necessarily sum to row and column totals.

^b^ Includes American Indian or Alaska Native, Asian, multiracial, other, “don’t know,” and declined to answer.

### Birth Cohort Trends in Total Cholesterol Level

**Figure 2.**
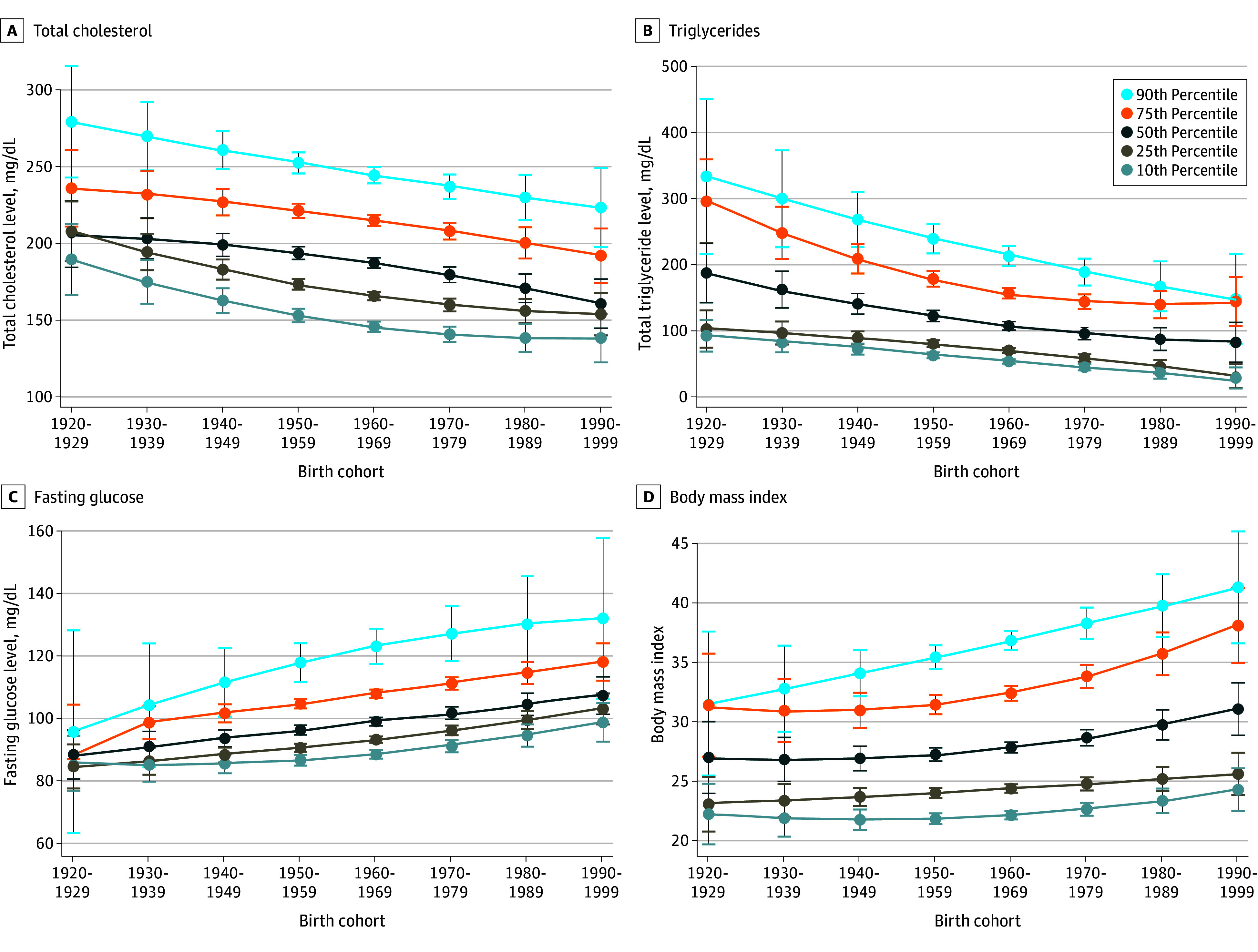
Adjusted Trends of Total Cholesterol, Fasting Triglyceride, and Fasting Glucose Levels and Body Mass Index (BMI) by Birth Cohort Data are from National Health and Nutrition Examination Survey (NHANES), 1999 to 2020. The curves are based on quantile regression models adjusted for sex, race (with non-Hispanic White race as the reference group), age, square of the age, square of the cohort, and age × cohort interaction. The models for total cholesterol level and BMI used NHANES mobile examination center subsample weights. The models for triglyceride and glucose levels used fasting subsample weights.

Unadjusted median total cholesterol level decreased from 200 (IQR, 173-277) mg/dL among the birth cohort born in the 1920s cohort to 166 (IQR, 147-189) mg/dL among the cohort born in the 1990s (eTable 2 in Supplement 1) (to convert to mmol/L, multiply by 0.0259). In adjusted models, total cholesterol level was lower in more recent (younger) birth cohorts compared with older birth cohorts in the 90th, 75th, 50th, 25th, and 10th percentiles of total cholesterol levels (Figure 2). For total cholesterol, the AMEs of birth cohort were −7.7 (95% CI, −9.5 to −6.0) mg/dL for the 90th percentile, −6.7 (95% CI, −7.9 to −5.5) mg/dL for the 75th percentile, −7.1 (95% CI, −8.2 to −6.1) mg/dL for the 50th percentile, −6.7 (95% CI, −7.6 to −5.7) mg/dL for the 25th percentile, and −6.0 (95% CI, −7.1 to −4.9) mg/dL for the 10th percentile (Table 2).

**Table 2.** Adjusted AME of Birth Cohort on Quantiles of Total Cholesterol, Fasting Triglyceride, and Fasting Glucose Levels and BMI

Outcome	AMEs (95% CI)^a^				
	90th percentile	75th percentile	50th percentile	25th percentile	10th percentile
Total cholesterol level, mg/dL	−7.7 (−9.5 to −6.0)	−6.7 (−7.9 to −5.5)	−7.1 (−8.2 to −6.1)	−6.7 (−7.6 to −5.7)	−6.0 (−7.1 to −4.9)
Fasting triglyceride level, mg/dL	−25.2 (−30.0 to −20.3)	−16.8 (−19.4 to −14.2)	−13.1 (−15.1 to −11.1)	−11.0 (−12.2 to −9.7)	−8.9 (−10.0 to −7.8)
Fasting glucose level, mg/dL	4.6 (2.6 to 6.6)	3.2 (2.7 to 3.8)	2.7 (2.3 to 3.1)	2.8 (2.5 to 3.2)	2.3 (1.9 to 2.7)
BMI	1.4 (1.1 to 1.8)	1.2 (1.0 to 1.4)	0.7 (0.6 to 0.9)	0.4 (0.3 to 0.5)	0.4 (0.3 to 0.5)

Abbreviations: AME, average marginal effect; BMI, body mass index (calculated as the weight in kilograms divided by the height in meters squared.

SI conversion factors: To convert cholesterol to mmol/L, multiply by 0.0259; glucose to mmol/L, multiply by 0.0555; triglyceride to mmol/L, multiply by 0.0113.

^a^ Represents the mean change in outcomes per 1-decade younger birth cohort, based on quantile regression models adjusted for sex, race (non-Hispanic White race was the reference group), age, square of age, square of cohort, and age × cohort interaction. The models for total cholesterol level and BMI used National Health and Nutrition Examination Survey mobile examination center subsample weights. The models for triglyceride and glucose levels used fasting subsample weights.

Table 3 showed that more recent birth cohorts had lower total cholesterol levels than older birth cohorts. The total effects of being born a decade later from 1920s to 1990s ranged from −13.1 (95% CI, −16.9 to −9.2) to −5.1 (95% CI, −6.6 to −3.5) mg/dL. For those who were born in and after the 1960s, the direct effects of birth cohort on total cholesterol levels were more pronounced than the total effects (1970s vs 1960s: −6.5 [95% CI, −8.0 to −4.9] mg/dL; 1980s vs 1970s: −10.7 [95% CI, −12.2 to −9.3] mg/dL; and 1990s vs 1980s: −7.3 [95% CI, −8.8 to −5.8] mg/dL), indicating a suppressive effect of BMI on the associations (indirect effects: 1970s vs 1960s: 0.5 [95% CI, 0.3-0.7] mg/dL; 1980s vs 1970s: 0.6 [95% CI, 0.4-0.8] mg/dL; and 1990s vs 1980s: 0.4 [95% CI, 0.2-0.7] mg/dL).

**Table 3.** Adjusted Total and Indirect Associations Between Birth Cohort and Total Cholesterol, Fasting Triglyceride, and Fasting Glucose Levels, Mediated via BMI^a^

Birth cohort	β (95% CI)		
	Total effect	Direct effect	Indirect effect
**Total cholesterol level, mg/dL**			
1930-1939 vs 1920-1929	−13.1 (−16.9 to −9.2)	−12.8 (−16.7 to −9.0)	−0.2 (−0.4 to −0.1)
1940-1949 vs 1930-1939	−9.0 (−11.0 to −6.9)	−8.4 (−10.5 to −6.3)	−0.6 (−0.8 to −0.3)
1950-1959 vs 1940-1949	−9.1 (−10.8 to −7.4)	−8.7 (−10.4 to −7.0)	−0.4 (−0.5 to −0.2)
1960-1969 vs 1950-1959	−5.1 (−6.6 to −3.5)	−5.0 (−6.5 to −3.4)	−0.1 (−0.2 to 0.0)
1970-1979 vs 1960-1969	−6.0 (−7.5 to −4.4)	−6.5 (−8.0 to −4.9)	0.5 (0.3 to 0.7)
1980-1989 vs 1970-1979	−10.2 (−11.6 to −8.7)	−10.7 (−12.2 to −9.3)	0.6 (0.4 to 0.8)
1990-1999 vs 1980-1989	−6.8 (−8.4 to −5.3)	−7.3 (−8.8 to −5.8)	0.4 (0.2 to 0.7)
**Fasting triglyceride level, mg/dL**			
1930-1939 vs 1920-1929	−17.8 (−28.6 to −6.9)	−18.7 (−29.6 to −7.7)	0.9 (0.2 to 1.5)
1940-1949 vs 1930-1939	−22.1 (−29.5 to −14.7)	−23.4 (−30.9 to −15.9)	1.3 (0.7 to 1.9)
1950-1959 vs 1940-1949	−23.6 (−30.6 to −16.5)	−25.0 (−32.0 to −17.9)	1.4 (0.7 to 2.1)
1960-1969 vs 1950-1959	−16.9 (−23.6 to −10.2)	−18.4 (−25.2 to −11.7)	1.5 (0.9 to 2.2)
1970-1979 vs 1960-1969	−12.3 (−19.1 to −5.6)	−16.3 (−23.1 to −9.6)	4.0 (3.0 to 5.1)
1980-1989 vs 1970-1979	−19.6 (−25.4 to −13.7)	−21.9 (−27.7 to −16.0)	2.3 (1.6 to 3.1)
1990-1999 vs 1980-1989	−20.0 (−24.9 to −15.2)	−21.5 (−26.3 to −16.8)	1.5 (0.8 to 2.2)
**Fasting glucose level, mg/dL**			
1930-1939 vs 1920-1929	5.1 (0.4 to 9.8)	4.6 (−0.1 to 9.4)	0.5 (0.1 to 0.8)
1940-1949 vs 1930-1939	3.6 (0.8 to 6.5)	3.0 (0.2 to 5.9)	0.6 (0.3 to 0.8)
1950-1959 vs 1940-1949	−0.1 (−2.4 to 2.2)	−0.8 (−3.1 to 1.5)	0.7 (0.4 to 1.0)
1960-1969 vs 1950-1959	4.6 (2.6 to 6.6)	4.0 (2.0 to 6.0)	0.6 (0.4 to 0.8)
1970-1979 vs 1960-1969	5.7 (3.9 to 7.5)	4.6 (2.8 to 6.4)	1.1 (0.8 to 1.4)
1980-1989 vs 1970-1979	4.0 (2.5 to 5.4)	3.4 (2.0 to 4.9)	0.5 (0.3 to 0.7)
1990-1999 vs 1980-1989	1.7 (0.2 to 3.2)	1.5 (0.0 to 3.0)	0.2 (0.1 to 0.3)

Abbreviation: BMI, body mass index (calculated as the weight in kilograms divided by the height in meters squared).

SI conversion factors: To convert cholesterol to mmol/L, multiply by 0.0259; glucose to mmol/L, multiply by 0.0555; triglyceride to mmol/L, multiply by 0.0113.

^a^ All results are based on causal mediation analysis using parametric regression models adjusted for sex, self-identified race and ethnicity (non-Hispanic White race was the reference group; race and ethnicity represent social constructs), age, and square of age.

### Birth Cohort Trends in Fasting Triglycerides

Unadjusted median triglyceride levels decreased from 126 (IQR, 93-173) mg/dL among the 1920s cohort to 71 (IQR, 50-105) mg/dL among the 1990s cohort (eTable 2 in Supplement 1) (to convert to mmol/L, multiply by 0.0113). In adjusted models, triglyceride levels were lower among younger birth cohorts in each percentile (Figure 2), with the AMEs for triglyceride level per decade younger birth cohort being −25.2 (95% CI, −30.0 to −20.3) mg/dL for the 90th percentile, −16.8 (95% CI, −19.4 to −14.2) mg/dL for the 75th percentile, −13.1 (95% CI, −15.1 to −11.1) mg/dL for the 50th percentile, −11.0 (95% CI, −12.2 to −9.7) mg/dL for the 25th percentile, and −8.9 (95% CI, −10.0 to −7.8) mg/dL for the 10th percentile (Table 2).

The adjusted differences in triglyceride levels between successive birth cohorts (total effects) are shown in Table 3. The total effects of birth cohorts across the study period ranged from −23.6 (95% CI, −30.6 to −16.5) to −12.3 (95% CI, −19.1 to −5.6) mg/dL. Across all birth cohorts, the direct effects of birth cohort on total cholesterol levels were slightly more pronounced than the total effects, ranging from −25.0 (95% CI, −32.0 to −17.9) to −16.3 (95% CI, −23.1 to −9.6) mg/dL, indicating a suppressive effect of BMI on the associations with indirect effects ranging from 0.9 (95% CI, 0.2-1.5) to 4.0 (3.0-5.1) mg/dL.

### Birth Cohort Trends in Fasting Glucose Level

The unadjusted median fasting glucose level decreased from 103 (IQR, 95-116) mg/dL among the 1920s cohort to 96 (IQR, 90-102) mg/dL among the 1990s cohort (eTable 2 in Supplement 1) (to convert to mmol/L, multiply by 0.0555). The adjusted median fasting glucose was higher across younger birth cohorts (Figure 2). This discrepancy was mainly due to the different age distributions within each cohort (eFigure in Supplement 1). Fasting glucose level was in general slightly higher among older cohorts. However, for any given age, younger cohort had slightly higher fasting glucose level (eFigure in Supplement 1). The AMEs for glucose level per decade younger cohort were 4.6 (95% CI, 2.6-6.6) for the 90th percentile, 3.2 (95% CI, 2.7-3.8) mg/dL for the 75th percentile, 2.7 (95% CI, 2.3-3.1) mg/dL for the 50th percentile, 2.8 (95% CI, 2.5-3.2) mg/dL for the 25th percentile, and 2.3 (95% CI, 1.9-2.7) mg/dL for the 10th percentile (Table 2).

Total effects comparing glucose levels in the birth cohort born a decade earlier ranged from 1.7 (95% CI, 0.2-3.2) to 5.7 (95% CI, 3.9-7.5) mg/dL, except that the difference between 1950s and 1940s was insignificant. Across birth cohorts, the direct effects of birth cohort were smaller than the total effects, ranging from 1.5 (95% CI, 0-3.0) to 4.6 (95% CI, 2.8-6.4) mg/dL, indicating BMI contributed to the associations (Table 3).

### Supplementary Analysis

Patterns of change (eTable 2 in Supplement 1) and AMEs (eTable 3 in Supplement 1) for non-HDL cholesterol levels across birth cohorts were similar to those observed for total cholesterol level. The results from the mediation analysis also showed that the increase in BMI potentially suppressed the favorable trends in non-HDL cholesterol levels in more recent birth cohorts (eTable 4 in Supplement 1). Total effects ranged from −9.7 (95% CI, −11.2 to −8.3) to −4.9 (95% CI, −6.5 to −3.4) mg/dL and indirect effects from 0.2 (95% CI, 0-0.3) to 1.5 (95% CI, 1.2-1.8) mg/dL. After additionally adjusting for statin use, the AMEs of birth cohort slightly decreased but were still statistically significant for all lipid outcomes (eTable 3 in Supplement 1). We did not find any moderating effect of the change in the guideline for therapy to lower lipid levels after 2013. After adjusting for statin use, all direct and indirect effects were halved for total cholesterol and non-HDL cholesterol outcomes, but the mediation pattern persisted (eTable 5 in Supplement 1).

## Discussion

In this cross-sectional analysis of nonpregnant US adults born between 1920 and 1999 who were examined between 1999 and 2020, more recent birth cohorts (or younger generations) had lower total cholesterol and fasting triglyceride levels and higher fasting glucose levels after adjusting for age, sex, and race and ethnicity. Population-level improvements in total cholesterol and triglyceride levels decelerated and adverse trends in glucose levels accelerated in more recent birth cohorts, which was mediated by changes in concurrent increases in BMI.

This study extends available data on secular trends in cardiometabolic health in the US. Previous research has examined secular trends or period effects in cholesterol, triglyceride, and glucose levels,^3,6,8,13,14^ which are complementary to changes or trends observed across birth cohorts or generations. While we found favorable changes with lower total cholesterol and triglyceride levels across birth cohorts, improvements have decelerated in more recent generations. The supplementary analysis supported that statin use had contributed significantly to the decreasing trends in lipid outcomes over the years. We did not find evidence for a differential cohort effect before and after the 2013 change in guidelines for therapy to lower lipid levels. In addition, glucose levels have continued to increase in more recent birth cohorts. These results align with previous research demonstrating differences in period effects in these cardiometabolic risk factor levels across time by age. Specifically, data from the 1999-2018 NHANES^4^ have demonstrated lower relative decreases in total cholesterol level among the group aged 20 to 39 years (5.5%) compared with the group 60 years or older (14.6%) and higher relative increases in diabetes prevalence among the group aged 20 to 39 years (48.5%) compared with the group 60 years or older (27.9%).

This study additionally quantifies the extent to which changes in BMI may be associated with changes in cholesterol, triglyceride, and glucose levels across different generations. Although BMI contributed to a significant proportion of the adverse trends in total cholesterol, triglyceride, and glucose levels in recent generations and should continue to be a focus of public health initiatives, up to 80% of the birth cohort effects were not mediated through BMI. This suggests the need for additional research to identify the behavioral and environmental factors that may be contributing to the observed birth cohort effect, as well as to inform preventive strategies tailored to specific birth cohorts independent of obesity.^16^ Potential contributors include higher dietary intake of saturated fats and simple carbohydrates,^29^ lower levels of physical activity, and higher levels of psychosocial stressors.^30,31^ Policies aimed at promoting both healthy lifestyle behaviors and reducing obesity are needed to address trends in cardiovascular disease risk factors among younger generations, such as education on healthy lifestyles in schools, regulation on the sale of partially hydrogenated oils, and sugary beverage taxes.^32^ Developing and promoting mobile health applications may be an innovative way to track nutrition and physical activity as well as deliver mental health services that could be uniquely effective in engaging younger generations.^32,33,34,35^

### Limitations

Limitations of this study include use of a serial cross-sectional analysis with lack of repeated measurements in the same participants. Therefore, we were unable to assess individual-level changes in cardiovascular risk factors over time. In addition, the causal mediation program does not currently support sampling weight. The mediation results cannot be generalized to the US population. While our evidence supported that statin use was significantly associated with the declining trends in lipid outcomes, the purpose of our analysis was mainly focused on quantifying birth cohort effect and was not able to sufficiently delineate the effect of statin use and other treatments to lower lipid levels. It is worth noting that use of statins and other drugs to lower lipid levels is very much correlated with age and birth cohort, as well as lipid outcomes. Therefore, results may have been attenuated after controlling for statin use, partially due to overadjustment. Finally, our analysis was limited to US adults. Future work is needed to determine the mediating effect of obesity on trends in cholesterol, triglyceride, and glucose levels in underage populations.

## Conclusions

In this serial, cross-sectional study of 52 006 participants representing 264 664 915 US adults born between 1920 and 1999, we found that more recent birth cohorts have lower levels of total cholesterol and fasting triglyceride levels, but higher levels of fasting glucose. Higher BMI in more recent birth cohorts was associated with deceleration in favorable changes in total cholesterol and triglyceride levels and acceleration in adverse increases in glucose levels. While the mean lipid profile in younger generations was improved compared with older generations, this improvement hinges on an ongoing push to continue to educate the public and health care professionals about modifying risk even at a young age. Public health initiatives that target antecedent health behaviors are needed to improve cardiometabolic health across generations.

## Notes

Supplement 1. **eTable 1.** Weighted Estimates of Eligible US Adults by Birth Cohort and Age **eTable 2.** Percentiles of Total Cholesterol, Non-HDL Cholesterol, Fasting Triglyceride, and Fasting Glucose Levels by Birth Cohorts **eTable 3.** Adjusted Average Marginal Effect (AMEs) of Birth Cohort on Quantiles of Non-HDL Cholesterol, Total Cholesterol, and Triglyceride Levels **eTable 4.** Adjusted Total and Indirect Associations Between Birth Cohort and Non-HDL Cholesterol, Mediated via Body Mass Index **eTable 5.** Adjusted Total and Indirect Associations Between Birth Cohort and Non-HDL Cholesterol, Total Cholesterol, and Fasting Triglyceride Levels, Mediated via Body Mass Index (Additionally Controlled for Statin Use) **eFigure.** Adjusted Percentile Curves as a Function of Age for Total Cholesterol, Fasting Triglyceride, and Fasting Glucose Levels by Birth Cohorts (1920-1999): NHANES 1999-2020

Supplement 2.
Data Sharing Statement

## References

[1] Martin SS, Aday AW, Almarzooq ZI, ; American Heart Association Council on Epidemiology and Prevention Statistics Committee and Stroke Statistics Subcommittee. 2024 Heart Disease and Stroke Statistics: a Report of US and global data from the American Heart Association. Circulation. 2024;149(8):e347-e913. doi:10.1161/CIR.0000000000001209 38264914 PMC12146881

[2] Arnett DK, Blumenthal RS, Albert MA, . 2019 ACC/AHA guideline on the primary prevention of cardiovascular disease: a report of the American College of Cardiology/American Heart Association Task Force on Clinical Practice Guidelines. Circulation. 2019;140(11):e596-e646. doi:10.1161/CIR.0000000000000678 30879355 PMC7734661

[3] Rosinger A, Carroll MD, Lacher D, Ogden C. Trends in total cholesterol, triglycerides, and low-density lipoprotein in US adults, 1999-2014. JAMA Cardiol. 2017;2(3):339-341. doi:10.1001/jamacardio.2016.4396 27902824 PMC7388068

[4] He J, Zhu Z, Bundy JD, Dorans KS, Chen J, Hamm LL. Trends in cardiovascular risk factors in US adults by race and ethnicity and socioeconomic status, 1999-2018. JAMA. 2021;326(13):1286-1298. doi:10.1001/jama.2021.15187 34609450 PMC8493438

[5] Carroll MD, Kit BK, Lacher DA. Trends in elevated triglyceride in adults: United States, 2001-2012. NCHS Data Brief 198. May 2015. Accessed December 1, 2023. https://www.cdc.gov/nchs/data/databriefs/db198.pdf 25973997

[6] Gao Y, Shah LM, Ding J, Martin SS. US trends in cholesterol screening, lipid levels, and lipid-lowering medication use in US adults, 1999 to 2018. J Am Heart Assoc. 2023;12(3):e028205. doi:10.1161/JAHA.122.028205 36625302 PMC9973640

[7] Fang M, Wang D, Coresh J, Selvin E. Trends in diabetes treatment and control in US adults, 1999-2018. N Engl J Med. 2021;384(23):2219-2228. doi:10.1056/NEJMsa2032271 34107181 PMC8385648

[8] Wang L, Li X, Wang Z, . Trends in prevalence of diabetes and control of risk factors in diabetes among US adults, 1999-2018. JAMA. 2021;326(8):1-13. doi:10.1001/jama.2021.9883 34170288 PMC8233946

[9] Mitchell NS, Catenacci VA, Wyatt HR, Hill JO. Obesity: overview of an epidemic. Psychiatr Clin North Am. 2011;34(4):717-732. doi:10.1016/j.psc.2011.08.005 22098799 PMC3228640

[10] Hales CM, Carroll MD, Fryar CD, Ogden CL. Prevalence of obesity and severe obesity among adults. NCHS Data Brief 360. February 2020. Accessed December 1, 2023. https://www.cdc.gov/nchs/data/databriefs/db360-h.pdf 32487284

[11] Powell-Wiley TM, Poirier P, Burke LE, ; American Heart Association Council on Lifestyle and Cardiometabolic Health; Council on Cardiovascular and Stroke Nursing; Council on Clinical Cardiology; Council on Epidemiology and Prevention; and Stroke Council. Obesity and cardiovascular disease: a scientific statement from the American Heart Association. Circulation. 2021;143(21):e984-e1010. doi:10.1161/CIR.0000000000000973 33882682 PMC8493650

[12] National Center for Health Statistics. National Health and Nutrition Examination Survey (NHANES). Reviewed September 24, 2024. Accessed December 1, 2023. https://www.cdc.gov/nchs/nhanes.htm

[13] Carroll MD, Kit BK, Lacher DA, Shero ST, Mussolino ME. Trends in lipids and lipoproteins in US adults, 1988-2010. JAMA. 2012;308(15):1545-1554. doi:10.1001/jama.2012.13260 23073951

[14] Mercado CI, Gregg E, Gillespie C, Loustalot F. Trends in lipid profiles and descriptive characteristics of US adults with and without diabetes and cholesterol-lowering medication use—National Health and Nutrition Examination Survey, 2003-2012, United States. PLoS One. 2018;13(3):e0193756. doi:10.1371/journal.pone.0193756 29509776 PMC5839584

[15] Goff DC, Howard G, Russell GB, Labarthe DR. Birth cohort evidence of population influences on blood pressure in the United States, 1887-1994. Ann Epidemiol. 2001;11(4):271-279. doi:10.1016/S1047-2797(00)00224-6 11306346

[16] Goff DC Jr, Labarthe DR, Howard G, Russell GB. Primary prevention of high blood cholesterol concentrations in the United States. Arch Intern Med. 2002;162(8):913-919. doi:10.1001/archinte.162.8.913 11966343

[17] Carroll MD, Lacher DA, Sorlie PD, . Trends in serum lipids and lipoproteins of adults, 1960-2002. JAMA. 2005;294(14):1773-1781. doi:10.1001/jama.294.14.1773 16219880

[18] Perak AM, Ning H, Kit BK, . Trends in levels of lipids and apolipoprotein B in US youths aged 6 to 19 years, 1999-2016. JAMA. 2019;321(19):1895-1905. doi:10.1001/jama.2019.4984 31112258 PMC6537842

[19] Wilson PWF, Jacobson TA, Martin SS, . Lipid measurements in the management of cardiovascular diseases: practical recommendations a scientific statement from the National Lipid Association Writing Group. J Clin Lipidol. 2021;15(5):629-648. doi:10.1016/j.jacl.2021.09.046 34802986

[20] National Health and Nutrition Examination Survey. Anthropometry procedures manual. Revised December 2000. Accessed December 1, 2023. https://wwwn.cdc.gov/nchs/data/nhanes/1999-2000/manuals/bm.pdf

[21] Goff DC Jr, Gillespie C, Howard G, Labarthe DR. Is the obesity epidemic reversing favorable trends in blood pressure? evidence from cohorts born between 1890 and 1990 in the United States. Ann Epidemiol. 2012;22(8):554-561. doi:10.1016/j.annepidem.2012.04.021 22683025

[22] Perraillon MC. Interpreting Model Estimates: Marginal Effects. Nova School of Business and Economics Lisbon; 2019.

[23] VanderWeele T. Explanation in Causal Inference: Methods for Mediation and Interaction. Oxford University Press; 2015.

[24] Pearl J. Interpretation and identification of causal mediation. Psychol Methods. 2014;19(4):459-481. doi:10.1037/a0036434 24885338

[25] Baron RM, Kenny DA. The moderator-mediator variable distinction in social psychological research: conceptual, strategic, and statistical considerations. J Pers Soc Psychol. 1986;51(6):1173-1182. doi:10.1037/0022-3514.51.6.1173 3806354

[26] Lee H, Cashin AG, Lamb SE, ; AGReMA group. A guideline for reporting mediation analyses of randomized trials and observational studies: the AGReMA Statement. JAMA. 2021;326(11):1045-1056. doi:10.1001/jama.2021.14075 34546296 PMC8974292

[27] Dahabreh IJ, Bibbins-Domingo K. Causal inference about the effects of interventions from observational studies in medical journals. JAMA. 2024;331(21):1845-1853. doi:10.1001/jama.2024.7741 38722735

[28] *Stata Statistical Software*. Release 17. StataCorp LLC; 2021. Accessed December 1, 2023. https://www.stata.com/stata17/

[29] Ushula TW, Mamun AA, Darssan D, . Dietary patterns and the risk of abnormal blood lipids among young adults: a prospective cohort study. Nutrition, metabolism, and cardiovascular diseases. Nutr Metab Cardiovasc Dis. 2022;32(5):1165-1174. doi:10.1016/j.numecd.2022.01.030 35260316

[30] Zhang Y, Yang J, Ye J, . Separate and combined associations of physical activity and obesity with lipid-related indices in non-diabetic and diabetic patients. Lipids Health Dis. 2019;18(1):49. doi:10.1186/s12944-019-0987-6 30755212 PMC6371482

[31] Chen DTL, Chen JCY, Chang JPC, Su KP. Lipids and mental health. In: Li D, ed. Advances in Dietary Lipids and Human Health. Academic Press; 2022:51-73. doi:10.1016/B978-0-12-823914-8.00021-5

[32] Brown T, Moore TH, Hooper L, . Interventions for preventing obesity in children. Cochrane Database Syst Rev. 2019;7(7):CD001871. 31332776 10.1002/14651858.CD001871.pub4 PMC6646867

[33] Bhardwaj NN, Wodajo B, Gochipathala K, Paul DP III, Coustasse A. Can mHealth revolutionize the way we manage adult obesity? Perspect Health Inf Manag. 2017;14(Spring):1a. 28566984 PMC5430129

[34] Chaddha A, Robinson EA, Kline-Rogers E, Alexandris-Souphis T, Rubenfire M. Mental health and cardiovascular disease. Am J Med. 2016;129(11):1145-1148. doi:10.1016/j.amjmed.2016.05.018 27288855

[35] Tevie J, Shaya FT. Association between mental health and comorbid obesity and hypertension among children and adolescents in the US. Eur Child Adolesc Psychiatry. 2015;24(5):497-502. doi:10.1007/s00787-014-0598-8 25146327

