# Supplementary material for: Cholesterol, Triglyceride, and Glucose Levels Across Birth Cohorts in the US
Source: JAMA Netw Open. 2024 Dec 6;7(12):e2449481. doi: 10.1001/jamanetworkopen.2024.49481 (PMC11624586; doi:10.1001/jamanetworkopen.2024.49481)
Supplement: Supplement 1. — eTable 1. Weighted Estimates of Eligible US Adults by Birth Cohort and Age eTable 2. Percentiles of Total Cholesterol, Non-HDL Cholesterol, Fasting Triglyceride, and Fasting Glucose Levels by Birth Cohorts eTable 3. Adjusted Average Marginal Effect (AMEs) of Birth Cohort on Quantiles of Non-HDL Cholesterol, Total Cholesterol, and Triglyceride Levels eTable 4. Adjusted Total and Indirect Associations Between Birth Cohort and Non-HDL Cholesterol, Mediated via Body Mass Index eTable 5. Adjusted Total and Indirect Associations Between Birth Cohort and Non-HDL Cholesterol, Total Cholesterol, and Fasting Triglyceride Levels, Mediated via Body Mass Index (Additionally Controlled for Statin Use) eFigure. Adjusted Percentile Curves as a Function of Age for Total Cholesterol, Fasting Triglyceride, and Fasting Glucose Levels by Birth Cohorts (1920-1999): NHANES 1999-2020 [file jamanetwopen-e2449481-s001.pdf]

Supplementary Online Content

Huang X, Petito LC, Shah NS, Lloyd-Jones DM, Khan SS, Cameron NA. Cholesterol, triglyceride, and glucose levels across birth cohorts in the US. *JAMA Netw Open*. 2024;7(12):e2449481. doi:10.1001/jamanetworkopen.2024.49481

eTable 1. Weighted Estimates of Eligible US Adults by Birth Cohort and Age
eTable 2. Percentiles of Total Cholesterol, Non-HDL Cholesterol, Fasting Triglyceride, and Fasting Glucose Levels by Birth Cohorts
eTable 3. Adjusted Average Marginal Effects (AMEs) of Birth Cohort on Quantiles of Non-HDL Cholesterol, Total Cholesterol, and Triglyceride Levels
eTable 4. Adjusted Total and Indirect Associations Between Birth Cohort and Non-HDL Cholesterol, Mediated via Body Mass Index
eTable 5. Adjusted Total and Indirect Associations Between Birth Cohort and Non-HDL Cholesterol, Total Cholesterol, and Fasting Triglyceride Levels, Mediated via Body Mass Index (Additionally Controlled for Statin Use)
eFigure. Adjusted Percentile Curves as a Function of Age for Total Cholesterol, Fasting Triglyceride, and Fasting Glucose Levels by Birth Cohorts (1920-1999): NHANES 1999-2020

This supplementary material has been provided by the authors to give readers additional information about their work.

© 2024 Huang X et al. *JAMA Network Open*.

eTable 1. Weighted Estimates of Eligible US Adults by Birth Cohort and Age[a]

	Age groups							
	**18-24**	**25-34**	**35-44**	**45-54**	**55-64**	**65-74**	**75-85+**	**Total**
**Overall**	28,495,394	46,615,437	48,917,412	50,460,531	42,529,260	28,995,046	18,651,834	264,664,915
**By birth cohorts**								
**1920-29**	0	0	0	0	0	899,815	5,025,849	5,925,664
**1930-39**	0	0	0	0	1,178,863	7,998,557	7,081,177	16,258,597
**1940-49**	0	0	0	2,059,771	11,682,954	12,835,539	6,544,808	33,123,071
**1950-59**	0	0	2,349,309	18,073,781	19,418,067	7,261,136	0	47,102,291
**1960-69**	0	2,189,183	18,241,310	21,831,606	10,249,377	0	0	52,511,476
**1970-79**	1,951,384	14,847,472	19,864,586	8,495,373	0	0	0	45,158,816
**1980-89**	12,187,682	20,505,719	8,462,208	0	0	0	0	41,155,609
**1990-99**	14,356,327	9,073,063	0	0	0	0	0	23,429,390

[a] The table shows the estimates of eligible US populations by age and birth cohort based on the National Health and Nutrition Examination Survey (NHANES) sample and Mobile Exam Center (MEC) sampling weights.

© 2024 Huang X et al. *JAMA Network Open*.

eTable 2. Percentiles of Total Cholesterol, Non-HDL Cholesterol, Fasting Triglyceride, and Fasting Glucose Levels by Birth Cohorts

		Birth cohorts							
**Outcomes**	**Overall**	**1920-29**	**1930-39**	**1940-49**	**1950-59**	**1960-69**	**1970-79**	**1980-89**	**1990-99**
**Total Cholesterol (mg/dL)**									
90th percentile	247	256	253	255	255	254	243	225	214
75th percentile	219	227	226	225	227	226	217	202	189
50th percentile	191	200	196	196	200	198	190	178	166
25th percentile	165	173	167	166	174	174	167	156	147
10th percentile	144	151	143	142	151	153	148	139	130
**Non-HDL Cholesterol (mg/dL)**									
90th percentile	200	196	199	202	203	193	178	167	200
75th percentile	169	169	168	172	173	167	152	137	169
50th percentile	144	140	138	145	144	138	125	113	144
25th percentile	118	113	111	119	119	114	102	93	118
10th percentile	98	91	90	97	99	95	85	76	98
**Fasting Triglycerides (mg/dL)**									
90th percentile	221	243	232	233	229	235	217	193	160
75th percentile	152	173	177	162	160	159	150	133	105
50th percentile	102	126	122	111	112	106	98	89	71
25th percentile	70	93	88	79	78	73	67	61	50
10th percentile	51	72	66	59	58	55	50	44	37
**Fasting Glucose (mg/dL)**									
90th percentile	125	138	148	143	134	123	116	110	108
75th percentile	108	116	120	117	113	108	105	102	102
50th percentile	99	103	105	105	102	100	97	96	96
25th percentile	92	95	96	96	95	93	91	90	90
10th percentile	86	90	90	90	89	87	85	85	86

All statistics are weighted to represent the eligible US population of 264,664,915.
P-value<0.001 based on Jonckheere-Terpstra test for all trends

© 2024 Huang X et al. *JAMA Network Open*.

eTable 3. Adjusted Average Marginal Effects (AMEs) of Birth Cohort on Quantiles of Non-HDL Cholesterol, Total Cholesterol, and Triglyceride Levels

	90th percentile		75th percentile		50th percentile		25th percentile		10th percentile	
**Outcomes**	**AME**	**95% CI**	**AME**	**95% CI**	**AME**	**95% CI**	**AME**	**95% CI**	**AME**	**95% CI**
**Not adjusted for Statin use**										
**Non-HDL Cholesterol (mg/dL)**	-8.9	(-10.7 to -7.1)	-8.3	(-9.5 to -7.0)	-7.9	(-8.8 to -6.9)	-7.2	(-8.2 to -6.3)	-6.4	(-7.5 to -5.3)
**Adjusted for Statin use**										
**Non-HDL Cholesterol (mg/dL)**	-7.9	(-9.7 to -6.1)	-7.1	(-8.3 to -5.8)	-6.6	(-7.7 to -5.6)	-5.7	(-6.6 to -4.7)	-5.2	(-6.2 to -4.1)
**Total Cholesterol (mg/dL)**	-7.0	(-8.9 to -5.2)	-5.7	(-6.8 to -4.6)	-5.6	(-6.6 to -4.6)	-5.0	(-5.9 to -4.2)	-3.7	(-4.7 to -2.8)
**Fasting Triglycerides (mg/dL)**	-26.4	(-31.4 to -21.5)	-17.8	(-20.7 to -14.9)	-13.1	(-15.0 to -11.2)	-11.3	(-12.7 to -9.9)	-9.1	(-10.3 to -8.0)

[a] The table shows AMEs and 95% CIs based on quantile regression models adjusted for gender, race (NH White as referent), age, age^2, cohort^2, and age x cohort interaction. The second panel additionally adjusted for Statin use. The models for total cholesterol and BMI used NHANES MEC subsample weights; The models for fasting triglycerides used fasting subsample weights.

© 2024 Huang X et al. *JAMA Network Open*.

eTable 4. Adjusted Total and Indirect Associations Between Birth Cohort and Non-HDL Cholesterol, Mediated via Body Mass Index

	Non-HDL Cholesterol (mg/dL)		
**Birth cohort**	**Total effect β (95% CI)**	**Direct effect β (95% CI)**	**Indirect effect β (95% CI)**
1930-39 vs. 1920-29	-13.1 (-17.0 to -9.3)	-13.2 (-17.1 to -9.3)	.1 (-0.0 to 0.2)
1940-49 vs. 1930-39	-10.0 (-12.1 to -7.9)	-9.9 (-12.0 to -7.8)	-.1 (-0.2 to 0.0)
1950-59 vs. 1940-49	-9.7 (-11.4 to -8.0)	-9.8 (-11.5 to -8.0)	.1 (-0.0 to 0.2)
1960-69 vs. 1950-59	-5.8 (-7.4 to -4.2)	-6.2 (-7.8 to -4.6)	.4 (0.2 to 0.5)
1970-79 vs. 1960-69	-5.5 (-7.1 to -4.0)	-7.0 (-8.5 to -5.4)	1.4 (1.1 to 1.8)
1980-89 vs. 1970-79	-9.7 (-11.1 to -8.2)	-10.9 (-12.3 to -9.4)	1.2 (0.8 to 1.5)
1990-99 vs. 1980-89	-6.8 (-8.4 to -5.2)	-7.7 (-9.2 to -6.2)	.9 (0.5 to 1.3)

[a] All results are based on causal mediation analysis using parametric regression models adjusted for gender, race (non-Hispanic White as referent), age, and age^2

© 2024 Huang X et al. *JAMA Network Open*.

eTable 5. Adjusted Total and Indirect Associations Between Birth Cohort and Non-HDL Cholesterol, Total Cholesterol, and Fasting Triglyceride Levels, Mediated via Body Mass Index (Additionally Controlled for Statin Use)

Birth cohort	Total effect β (95% CI)	Direct effect β (95% CI)	Indirect effect β (95% CI)
**Non-HDL Cholesterol (mg/dL)**			
1930-39 vs. 1920-29	-5.9 (-9.0 to -2.8)	-5.9 (-9.1 to -2.8)	.1 (-0.1 to 0.2)
1940-49 vs. 1930-39	-5.1 (-7.0 to -3.3)	-5.2 (-7.1 to -3.3)	.1 (-0.0 to 0.1)
1950-59 vs. 1940-49	-6.9 (-8.5 to -5.3)	-7.0 (-8.7 to -5.4)	.2 (0.0 to 0.3)
1960-69 vs. 1950-59	-4.9 (-6.5 to -3.4)	-5.3 (-6.9 to -3.7)	.4 (0.2 to 0.5)
1970-79 vs. 1960-69	-5.4 (-7.0 to -3.9)	-6.9 (-8.5 to -5.4)	1.5 (1.2 to 1.8)
1980-89 vs. 1970-79	-9.7 (-11.2 to -8.3)	-10.9 (-12.3 to -9.5)	1.2 (0.8 to 1.5)
1990-99 vs. 1980-89	-6.8 (-8.4 to -5.2)	-7.7 (-9.2 to -6.1)	.9 (0.5 to 1.3)
**Total Cholesterol (mg/dL)**			
1930-39 vs. 1920-29	-5.3 (-8.3 to -2.2)	-5.2 (-8.3 to -2.1)	.0 (-0.1 to 0.0)
1940-49 vs. 1930-39	-3.7 (-5.6 to -1.9)	-3.5 (-5.4 to -1.6)	-.2 (-0.4 to -0.1)
1950-59 vs. 1940-49	-6.0 (-7.6 to -4.3)	-5.8 (-7.4 to -4.2)	-.1 (-0.2 to 0.0)
1960-69 vs. 1950-59	-4.0 (-5.6 to -2.5)	-4.0 (-5.5 to -2.4)	.0 (-0.1 to 0.0)
1970-79 vs. 1960-69	-5.8 (-7.4 to -4.3)	-6.4 (-8.0 to -4.9)	.6 (0.4 to 0.8)
1980-89 vs. 1970-79	-10.2 (-11.7 to -8.8)	-10.8 (-12.3 to -9.4)	.6 (0.4 to 0.8)
1990-99 vs. 1980-89	-6.8 (-8.4 to -5.3)	-7.3 (-8.8 to -5.8)	.4 (0.2 to 0.7)
**Fasting Triglycerides (mg/dL)**			
1930-39 vs. 1920-29	-17.6 (-27.2 to -8.1)	-17.9 (-27.5 to -8.3)	.3 (-0.4 to 0.9)
1940-49 vs. 1930-39	-22.4 (-29.4 to -15.3)	-23.2 (-30.3 to -16.1)	.8 (0.3 to 1.4)
1950-59 vs. 1940-49	-24.2 (-31.2 to -17.2)	-25.2 (-32.2 to -18.2)	.9 (0.3 to 1.5)
1960-69 vs. 1950-59	-17.6 (-24.3 to -10.8)	-18.9 (-25.6 to -12.1)	1.3 (0.7 to 1.9)
1970-79 vs. 1960-69	-13.0 (-19.7 to -6.2)	-16.7 (-23.5 to -10.0)	3.8 (2.8 to 4.8)
1980-89 vs. 1970-79	-19.5 (-25.3 to -13.6)	-21.8 (-27.6 to -15.9)	2.3 (1.6 to 3.0)
1990-99 vs. 1980-89	-19.9 (-24.8 to -15.1)	-21.4 (-26.2 to -16.7)	1.5 (0.8 to 2.2)

[a] All results are based on causal mediation analysis using parametric regression models adjusted for gender, race (non-Hispanic White as referent), age, age^2, and statin use.

© 2024 Huang X et al. *JAMA Network Open*.

eFigure. Adjusted Percentile Curves as a Function of Age for Total Cholesterol, Fasting Triglyceride, and Fasting Glucose Levels by Birth Cohorts (1920-1999): NHANES 1999-2020

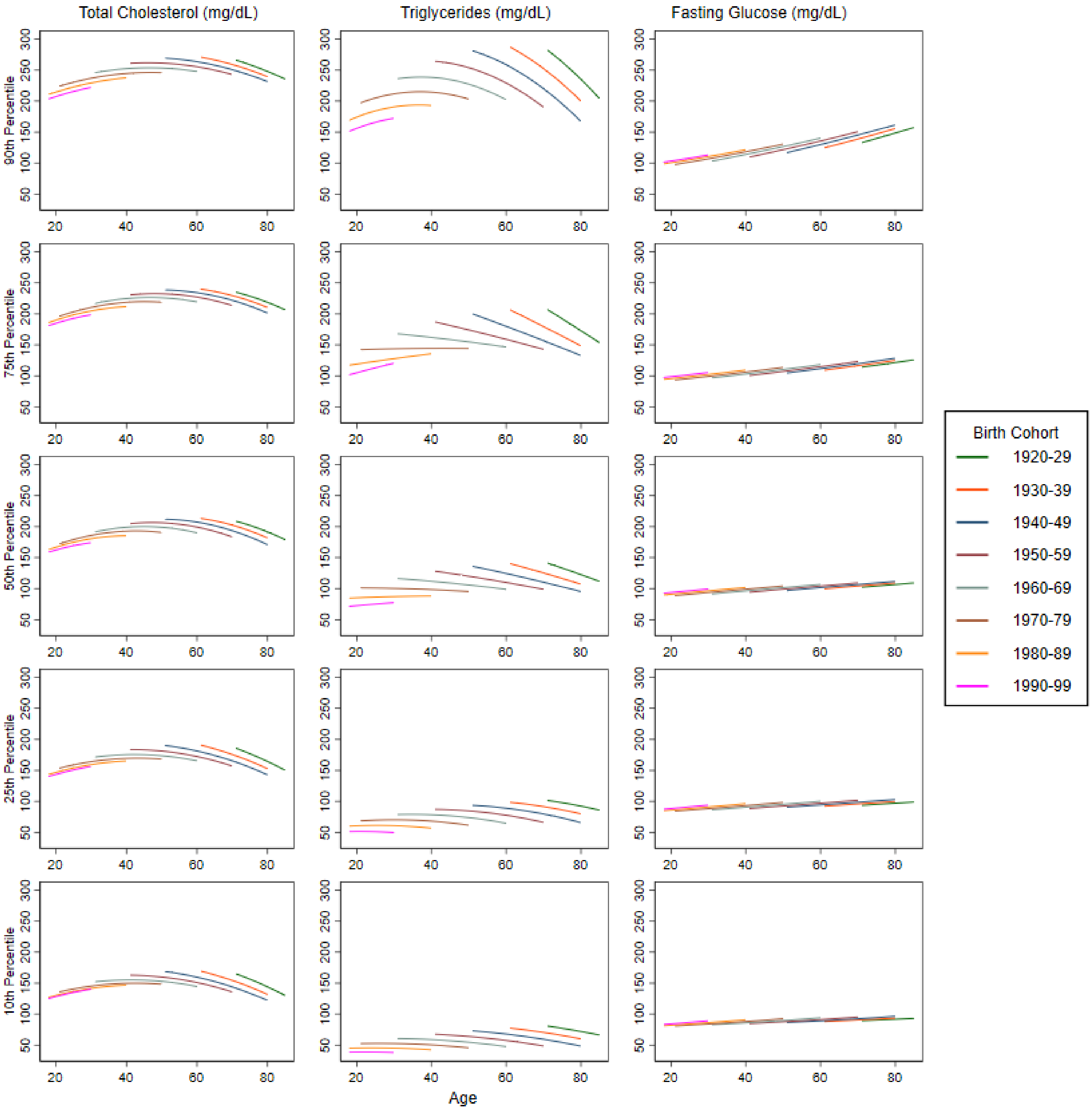

[a] The curves are based on quantile regression models adjusted for gender, race (NH White as referent), age, age^2, cohort^2, and age x cohort interaction. The models for total cholesterol and BMI used NHANES MEC subsample weights; The models for triglycerides and glucose used fasting subsample weights.

© 2024 Huang X et al. *JAMA Network Open*.
