# Supplementary material for: Cholesterol, Triglyceride, and Glucose Levels Across Birth Cohorts in the US
Source: JAMA Netw Open. 2024 Dec 6;7(12):e2449481. doi: 10.1001/jamanetworkopen.2024.49481 (PMC11624586; doi:10.1001/jamanetworkopen.2024.49481)
Supplement: Supplement 2. — Data Sharing Statement [file jamanetwopen-e2449481-s002.pdf]

Data Sharing Statement

Huang. Cholesterol, Triglyceride, and Glucose Levels Across Birth Cohorts in the US. *JAMA Netw Open*. Published December 06, 2024. doi:10.1001/jamanetworkopen.2024.49481

Data
Data available: Yes
Data types: Deidentified participant data
How to access data: https://wwwn.cdc.gov/nchs/nhanes/Default.aspx
When available: With publication

Supporting Documents
Document types: None

Additional Information
Who can access the data: The data is open to public.
Types of analyses: Available upon request.
Mechanisms of data availability: The data is open to public.
